# Editorial: Targeted cancer therapies, from small molecules to antibodies, volume II

**DOI:** 10.3389/fphar.2023.1147488

**Published:** 2023-06-30

**Authors:** Chao-Yue Su, Yan-Yan Yan, Jian-Ye Zhang, Yun-Kai Zhang, Zhe-Sheng Chen

**Affiliations:** ^1^ Department of Pharmacy, The Fifth Affiliated Hospital, Sun Yat-sen University, Zhuhai, Guangdong, China; ^2^ School of Medicine, Shanxi Datong University, Datong, China; ^3^ Guangzhou Municipal and Guangdong Provincial Key Laboratory of Molecular Target and Clinical Pharmacology, The NMPA and State Key Laboratory of Respiratory Disease, School of Pharmaceutical Sciences and the Fifth Affiliated Hospital, Guangzhou Medical University, Guangzhou, China; ^4^ BioSpatula LLC, Telford, PA, United States; ^5^ Department of Pharmaceutical Sciences, College of Pharmacy and Health Sciences, St. John’s University, New York, NY, United States

**Keywords:** cancer, target, small molecules, antibodies, therapy

Rapid advances in molecular biology have enabled an unprecedented understanding of the genes that drive tumorigenesis and progression, making innovative targeted therapy an attractive new strategy for treating cancers. By creating medications that target tumor-specific targets, such as small molecule drugs and monoclonal antibodies, the drug resistance and cytotoxicity of conventional chemotherapy drugs will be greatly reduced. Therefore, this Research Topic “*Targeted Cancer Therapies, from Small Molecules to Antibodies, Volume Ⅱ*” focuses on the role of novel targets and targeted anticancer drugs in reducing tumor therapy resistance and precise tumor therapy. To these ends, 32 articles, including 2 case reports, 10 reviews and 20 original research articles, were ultimately accepted for publication in this Research Topic.

The development of tumor resistance mechanisms is multifaceted and highly heterogeneous at the intratumoral or intercellular level. Due to clonal selection or evolution under therapeutic pressure, “off-target effects” frequently arise with a single tumor-targeting therapy, which may be related to novel genetic alterations in the targeted oncogene or other related oncogenes. Therefore, the combination of two targeted drugs is very necessary for tumor treatment. Wang et al. emphasized the use of dual-targeted therapy, that is, to deal with non-genetic drug resistance during tumor treatment by simultaneously inhibiting two or more related targets or pathways. The study by Wang et al. found that AZD5153, a small molecule inhibitor, targeting the DNA damage repair regulator MUS81, could enhance the antitumor efficacy of talazopanib. Mechanistically, AZD5153 impaired the activation of the ATR/CHK1 cell cycle signaling pathway and promoted gastric cancer cells with talazoparib-induced DNA damage to continue mitosis. Interestingly, a study performed by Fu et al. showed that antihypertensive drug amlodipine combined with gefitinib can synergistically inhibit the proliferation of non-small cell lung cancer (NSCLC) A549 cells by blocking the cell cycle. Similarly, Xia et al. uncovered that hypolipidemic drug simvastatin combined with DNA methyltransferase inhibitor 5-Aza-2’-deoxycytidine to mediate pyroptosis via GSDME (a pyroptotic effector) to exert anti-tumor effect. Li et al. showed that the combination of fruquintinib and sintilimab (anti-PD-1 antibody) greatly inhibits colorectal cancer growth by altering tumor immune microenvironment. Zhang et al. provided a case report, showing that the combination of the PARP inhibitor olaparib with EGFR TKI dacomitinib could significantly benefit a NSCLC patient with osimertinib-resistant brain and leptomeningeal metastases who had EGFR mutations combined with TP53 and ERBB2 mutations. In addition, double-dose icotinib exposure has also been shown to respond well to the treatment of targetable TKIs including almonertinib or osimertinib, in patients with emerging EGFR exon 20 T790M mutations (Chen et al.). Liu et al. showed that gemcitabine plus anlotinib was more effective and safe compared with gemcitabine plus docetaxel in advanced soft tissue sarcoma.

Additionally, several studies have shown that the drug delivery system could be used to enhance the active targeting capacity of drugs to improve the anti-tumor effect. For example, anti-CD47 antibody conjugated with valine-citrulline-monomethyl auristatin E (VCMMAE), named anti-CD47-VCMMAE, had a greater therapeutic effect on NSCLC tumors with high expression of *CD47* gene. Combining VCMMAE with anti-CD47 mAb was a novel and promising immunotherapy approach to directly kill NSCLC (Chiang et al.). Xie et al. highlighted the conjugation of chemotherapeutic agents to targeting ligands as a unique treatment approach for osteosarcoma. The selection of these targeting ligands was diverse, including antibodies, aptamers, peptides, saccharide, vitamin, bisphosphonates, hyaluronic acid and folate. Interestingly, Canals Hernaez et al. showed that PODO447 (a novel antibody targeting podocalyxin) conjugated to monomethyl auristatin E, could be used as a highly specific and potent antibody-drug conjugate to kill ovarian, pancreatic, glioblastoma, and leukemia cell lines. Therefore, PODO447-antibody drug conjugates served as precise tumor-specific and highly effective immunotherapeutic agents for targeting human tumors. In addition, Diao et al. prepared liposomes of peanut agglutinin-conjugated irinotecan hydrochloride and capecitabine with a stronger ability to target MUC1 (tumor-associated antigen mucin 1)-positive liver cancer cells.

Over the past decade, a great deal of research has been directed at RNA-based therapeutics. Therapeutic targeting of noncoding RNAs (ncRNAs), such as microRNAs (miRNAs) and long noncoding RNAs (lncRNAs), is propelling the future advancement of biomarker development, as well as represents an attractive approach for the treatment of human tumor. In those review articles, Fu and Xu summarized the emerging role of transfer RNA-derived small RNAs (tsRNAs) as cancer diagnostic and prognostic biomarkers. In addition, N6-methyladenosine (m6A), as a major internal epigenetic modification in eukaryotic mRNA, is a promising therapeutic target for malignant tumors. Zhu et al. introduced the molecular mechanism of m6A methylation involved in regulation and its role in tumor therapy. Wang et al. showed that silencing of lncRNA CHRM3-AS2 expression inhibited the malignant progression of glioma by regulating miR-370-5p/KLF4 expression. Another study showed that lncRNA TMPO-AS1 was overexpressed in endometrial cancer (EC) tissues and was closely related to the proliferation, invasion, glycolysis and paclitaxel resistance of EC cells. Dysregulation of lncRNA TMPO-AS1-miR-140/miR-143 axis promoted glycolysis and drug resistance in EC cells by upregulating GLUT1 expression (Dong et al.). It is worth noting that many recent studies have focused on the role of circular RNAs (circRNAs) in regulating gene transcription and splicing and encoding proteins. CircRNAs exert competitive endogenous RNA (ceRNA) to sponge miRNA and thus play an integral role in tumorigenesis. Shi et al. showed that silencing circOMA1 inhibits osteosarcoma progression by sponging miR-1294 to regulate c-Myc expression.

With the development of targeted therapy and immunotherapy, more and more cancer-related targets and signal transduction pathways have been examined. Designing and investigating small molecule inhibitors or antibodies by targeting these oncogenes or signaling pathways provides new opportunities for developing new therapeutic strategies in personalized oncology. For example, the c-Met gene is overexpressed in many human tumors and is related to the proliferation, differentiation, invasion and drug resistance of tumor cells. Anti-tumor therapies targeting c-Met, such as TKIs, monoclonal Antibodies and adoptive immunotherapy have shown remarkable effects in the treatment of digestive system tumors (Zhang et al.). Two review articles highlighted the crucial roles of the transmembrane proteins 88 (TMEM88) and 16A (TMEM16A) in tumorigenesis, progression, metastasis, proliferation and apoptosis, respectively (Guo et al.; Cai et al.). Therefore, inhibitors targeting TMEM88 and TMEM16A are expected to become new strategies in the treatment of malignant tumors. Wu et al. discovered a new potential therapeutic target for bladder cancer: vaccinia-related kinase 1, and confirmed that it could significantly inhibit the proliferation of bladder cancer cells *in vivo* and *in vitro*. Overexpression of SEMA3C played an important role in promoting cancer cell survival, which could be used as a new therapeutic target or diagnostic marker in pancreatic cancer, especially in tumors harboring specific KRAS G12D mutation. Zhang et al. discovered a gene named SEMA3C was highly expressed in pancreatic cancer cell lines and patients with a G12D mutation in KRAS. Of note, a case report performed by Luo et al. identified the expression of a novel *FGFR2-KIAA1217* fusion gene in gastrointestinal stromal tumors. PI3K/AKT is an important cancer-related pathway and designing new anti-tumor targeted drugs by targeting this pathway is a very promising therapeutic prospect. Peng et al. reviewed that aberrant activation of the PI3K/AKT/mTOR pathway has a significant role in carcinogenesis. Guo et al. showed that hinokiflavone, a natral double flavone compound, inhibits the growth of esophageal squamous cell carcinoma by regulating PI3K/AKT/mTOR signal pathway to induce apoptosis. Hinokiflavone can be used as a complementary/alternative agent for ESCC therapy. Another similar study also highlighted the importance of targeting the PI3K/AKT signaling pathway in tumor therapy. Vemurafenib suppresses breast cancer by targeting PI3K/AKT signaling pathway to inhibit immune evasion biomarker BCL2A1 (Dai et al.).

Recent studies have found that epigenetic enzyme-mediated transcriptional dysregulation of oncogenes or tumor suppressor genes is closely related to the occurrence, progression and prognosis of tumors. Small molecule compounds targeting epigenetic regulatory enzymes such as DNA methylases, histone modifiers (methylation and acetylation), enzymes that specifically recognize post-translational modifications, and post-transcriptional regulators have emerged as promising therapeutic approaches (Jin et al.). Ma et al. showed that histone deacetylase inhibitor I13 could significantly inhibit proliferation and colony formation of acute myeloid leukemia cells by inducing cell differentiation coupled with cell-cycle exit at G0/G1. It had also been shown that the natural product pectolinarigenin inhibited the proliferation of glioblastoma by inhibiting ribonucleotide reductase subunit M2 to induce cell cycle arrest (Jiang et al.). In addition, Guo et al. highlighted the critical role of adenylyl cyclase, a protease superfamily associated with cancer drug resistance and progression, in mediating tumor drug resistance and prognostic therapeutic targets. What’s more, developing anticancer compounds that could inhibit DNA or RNA repair enzymes was also a promising approach to fighting cancer. Kirsanov et al. found that 7-methylguanine (7-MG) could competitively inhibit DNA repair enzyme poly (ADP-ribose) polymerase and RNA-modifying enzyme tRNA-guanine transglycosylase and represents a potential anticancer drug candidate. Xing et al. designed a novel compound targeting aminopeptidase N (a type II membrane bound metalloprotease, also known as CD13), which could be used as a tumor chemosensitizer and a lead compound for cancer stem cell-based therapy.

In summary, the research topic of “*Targeted Cancer Therapies, from Small Molecules to Antibodies, Volume Ⅱ*” emphasized the significance of both new small molecules and antibodies in cancer targeting therapy. With a deeper understanding of oncogenes or tumor suppressor genes and the elucidation of tumor-related signal transduction pathways, we anticipated that more and more anticancer small molecules and antibodies will be developed, opening up a new avenue of precise cancer therapy.

